# Chemical Compositions, Chromatographic Fingerprints and Antioxidant Activities of Andrographis Herba

**DOI:** 10.3390/molecules191118332

**Published:** 2014-11-10

**Authors:** Yang Zhao, Chun-Pin Kao, Kun-Chang Wu, Chi-Ren Liao, Yu-Ling Ho, Yuan-Shiun Chang

**Affiliations:** 1 Department of Chinese Pharmaceutical Sciences and Chinese Medicine Resources, College of Pharmacy, China Medical University, Taichung 40402, Taiwan; 2 Department of Nursing, Hsin Sheng College of Medical Care and Management, Taoyuan 32544, Taiwan; 3 Department of Nursing, Hungkuang University, Taichung 43302, Taiwan; 4 Chinese Crude Drug Pharmacy, China Medical University Hospital, Taichung 40402, Taiwan

**Keywords:** Andrographis Herba, quantitative determination, chromatographic fingerprint, antioxidant activities, correlation analysis

## Abstract

This paper describes the development of an HPLC-UV-MS method for quantitative determination of andrographolide and dehydroandrographolide in Andrographis Herba and establishment of its chromatographic fingerprint. The method was validated for linearity, limit of detection and quantification, inter- and intra-day precisions, repeatability, stability and recovery. All the validation results of quantitative determination and fingerprinting methods were satisfactory. The developed method was then applied to assay the contents of andrographolide and dehydroandrographolide and to acquire the fingerprints of all the collected Andrographis Herba samples. Furthermore, similarity analysis and principal component analysis were used to reveal the similarities and differences between the samples on the basis of the characteristic peaks. More importantly, the DPPH free radical-scavenging and ferric reducing capacities of the Andrographis Herba samples were assayed. By bivariate correlation analysis, we found that six compounds are positively correlated to DPPH free radical scavenging and ferric reducing capacities, and four compounds are negatively correlated to DPPH free radical scavenging and ferric reducing capacities.

## 1. Introduction 

Andrographis Herba (AH), the aerial part of *Andrographis paniculata* (Burm. F.) Nees, has been widely used for clearing heat and removing toxicity, cooling blood and detumescence [1]. Due to its efficacy and affordability, the raw herb material is popular and is widely used in many Chinese herbal compound prescriptions. Modern pharmacological studies indicate that AH has various activities including antimicrobial and antioxidant [2], antimalarial [3], antiangiogenic [4], anti-inflammatory [5], anti-diabetic [6], and *in vitro* α-glucosidase and α-amylase enzyme inhibitory [7] activities. 

Andrographolides were demonstrated to be the active compounds of AH [8,9], showing manifold activities [10,11,12,13,14,15]. Andrographolide and dehydroandrographolide are used as chemical markers for quality control of AH in the Chinese Pharmacopoeia (2010 edition) which stipulates that the total content of the two markers should not be less than 0.8% in qualified AH samples. Therefore, several quantitative determination methods using HPLC-UV and HPLC-MS have been reported for routine control of AH and AH-related commercial products [16,17,18,19,20]. However, as a complex matrix with multiple ingredients, on one hand, the quality of an herb cannot be evaluated well by quantification of several chemical compounds, while on the other hand, it is hard to see which single compound might be responsible for the overall efficacy of the herb [21]. 

Fingerprinting analysis has been introduced and accepted by the World Health Organization (WHO) as a strategy for assessing the quality of herbal medicines [22,23,24] and plays a very important role in their development of modernization. Nonetheless, most studies on fingerprints of herbal medicines mainly aim to describe their chemical profiles. The correlation between chemical compounds and therapeutic effects of an herb is usually not elaborated. 

Based on this idea, a chromatographic fingerprinting method using HPLC-UV-MS was developed in the present study. On the one hand, the contents of andrographolide and dehydroandrographolide were quantified based on the values obtained by UV detection. On the other hand, the characteristic peaks were characterized based on the MS data. Furthermore, similarity analysis and principal components analysis (PCA) were performed using the contents or PA/W (peak area divided by sample weight) values of the characteristic peaks as variables. Most importantly, antioxidant capacities of AH samples were tested and fingerprint-efficacy correlation was studied for the first time. 

## 2. Results and Discussion

### 2.1. Optimization of the Extraction Method

The extraction solvent was optimized taking the extraction efficiency of andrographolide and dehydroandrographolide as indexes. Ethanol and methanol with ultrasonic extraction at room temperature were investigated. AH powder (0.2 g) was extracted with ethanol or methanol (10 mL) three times (30 min each time). The contents of andrographolide and dehydroandrographolide obtained using methanol were both higher than those obtained using ethanol. Furthermore, the two compounds were almost extracted completely (>99%) the second time (Supplementary Figures S1 and S2). Therefore, the optimal extraction procedure as described in Section 3.2. Sample and reference preparation was established.

### 2.2. Optimization of Chromatographic Conditions

To develop a reliable chromatographic fingerprinting method, an optimized strategy for HPLC conditions was performed. To obtain sharp and symmetrical peaks, different mobile phase systems including methanol–water and acetonitrile–water elution systems were tested. As a result, good resolution, baseline, sharp and symmetrical peaks were obtained by using an acetonitrile–water system. A few different columns (Waters XTerra RP18, Thermo Ascentis C18 and Grace Alltima C18) were tested before Waters XBridge C18 column (250 mm × 4.6 mm i.d., 3.5 μm) was finally selected as the column of choice. To obtain a sufficiently large number of detectable peaks in the chromatographic fingerprints, PAD full scan (190–400 nm) was used to acquire all the main peaks and finally 225 nm was selected as detection wavelength. Different column temperatures at 20, 25, 30 and 35 °C were also investigated. Although chromatograms detected at different temperatures did not show obvious differences, 35 °C was selected as the preferable one in order to minimize the influences from room temperature on the chromatograms. In the process of gradient optimization, gradient time, gradient procedure and initial composition of the mobile phase were taken into consideration. Finally, the gradient procedure was optimized as described in Section 3.3. HPLC Analysis.

### 2.3. Validation of Quantitative Analytical Method

The HPLC method was validated by defining the limits of detection (LOD) and quantification (LOQ), linearity, inter-day and intra-day precisions, repeatability, stability, and recovery. The calibration curves were plotted on the basis of linear regression analysis of the integrated peak areas (*y*) *versus* concentrations (*x*, mg/L) of the two analytes at five different levels. LOD and LOQ values for each analyte under the present chromatographic conditions were determined in terms of baseline noise, according to the IUPAC definition. LOD was determined as the analyte concentration yielding signal with a single-to-noise (S/N) ratio at 3:1, whereas the LOQ was defined as the analyte concentration yielding signal with S/N ratio at 10:1.The results of regression equations, correlation coefficients, linear ranges, LODs and LOQs for andrographolide and dehydroandrographolide are shown in Table 1. The correlation coefficient (*R*^2^) of the regression equation for each analyte indicates good linearity, being better than 0.999. 

Intra- and inter-day variations were chosen to determine the precision of the developed method. For intra-day variability test, one of the mixed standard solutions (andrographolide, 125 μg/mL; dehydroandrographolide, 125 μg/mL) was analyzed five times within one day, while for inter-day variability, the mixed standard solution was examined in triplicate each day on three consecutive days. The RSDs for the peak areas were calculated as measurements of precisions. The RSDs of intra-day variation for andrographolide and dehydroandrographolide were less than 0.1%, and the RSDs of inter-day variation for the two analytes were less than 3.00%, as shown in Table 2. Repeatability was evaluated by analyzing five different working solutions prepared from the same sample (AH-01). RSD values were 1.13% and 1.33% for andrographolide and dehydroandrographolide, respectively (Table 2). Stability was determined using repeated analyses of the same sample solution at different times during storage at room temperature (approx. 25 °C) for 24 h. The RSD values of peak areas of andrographolide and dehydroandrographolide were 1.07% and 1.02%, respectively (Table 2), indicating that the stability of the sample solution within one day was satisfactory. Recovery test was determined using spiked AH samples. A portion of 0.1000 g of AH sample was individually spiked with 0.1000 mg of andrographolide and 0.8000 mg of dehydroandrographolide, respectively. Five replicate samples were extracted and analyzed according to the procedures described above. As shown in Table 3, the mean recovery (*n* = 5) are 107.02% ± 1.52% and 98.86% ± 1.46%, respectively. These RSD values indicate that the proposed methodology is reproducible and suitable for the quantitative determination of andrographolide and dehydroandrographolide in AH samples. 

### 2.4. Validation of the Chromatographic Fingerprinting Method

The chromatographic fingerprinting method was validated for its inter- and intra-day precisions, repeatability and stability. The inter-day precision was evaluated by analyzing five injections of the same testing sample consecutively within one day. The intra-day precision was evaluated by analyzing the same testing sample in triplicate each day for three consecutive days. The repeatability was examined by determining five different solutions prepared from the same botanical sample (AH-01). The stability test was performed by analyzing the same sample solution at different time points (0, 2, 4, 8, 16 and 24 h). The results are expressed in two forms. One is the RSDs of the relative retention times (RRT) and relative peak areas (RPA) of each characteristic peak to the reference peak (andrographolide, in the present study). The other is the similarity values expressed as correlation coefficient or angle cosin. As a result (Supplementary Tables S1 and S2 ), the RSD values are all less than 4.50% and the similarity values are all greater than 0.99, indicating that the established chromatographic fingerprinting method is satisfactory for fingerprint study.

### 2.5. Quantitative Determination of the Two Analytes in AH Samples

The developed HPLC-UV analytical method was applied for the quantitative determination of andrographolide and dehydroandrographolide in ten AH samples. The calibration curves were used to calculate the contents of the two compounds in AH samples (data are shown in Table 4). To begin with, the contents of andrographolide and dehydroandrographolide in different samples varied from 0.1122% to 1.5926%, 0.3991% to 1.3472%, respectively. AH-10 was found to have the highest content of andrographolide at 1.5926%, meanwhile, AH-03 had the highest content of dehydroandrographolide at 1.3472%. The lowest content of andrographolide was found in AH-08 at 0.1122%, meanwhile, the lowest content of dehydroandrographolide was found in AH-05 at 0.3991%. Then, the highest total content of andrographolide and dehydroandrographolide was found in AH-10 at 2.4083%, conversely, AH-08 gave the lowest one at 0.5317%. Besides AH-10, three other AH samples, AH-01, AH-03 and AH-06, had the total content of the two analytes above 2.0%. Finally, according to the specification in CP (edition 2010), AH-05 and AH-08 were unqualified herbs that could not be used clinically. 

### 2.6. Assignments of the Characteristic Peaks

**Figure 1:**
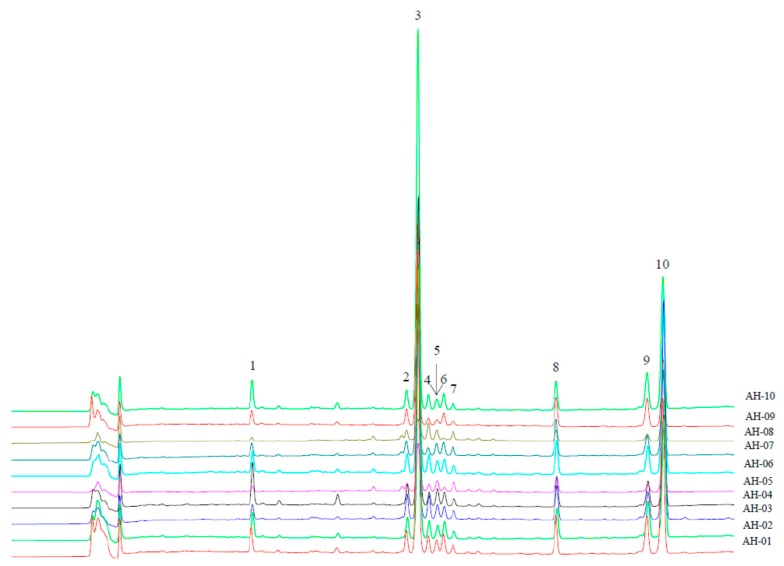
Overlapped chromatograms of AH samples detected at 225 nm.

Figure 1 shows the ten characteristic peaks of all the tested samples detected at 225 nm. The structural identification of each peak was performed on the basis of MS and MS^2^ experiments (Table 5). Under the optimized MS conditions, both negative and positive ESI modes were used in our experiment.

Peak 1 occurs at a retention time of 7.4 min with maximal UV absorption at 225 nm. In negative ion mode, a deprotonated molecular ion at *m/z* 511 [M−H]^−^, a formic acid adduct ion at *m/z* 557 [M−H+HCOOH]^−^ as well as an ion at *m/z* 493 [M−H−H_2_O]^−^ were found in its MS spectrum. Fragmentation of the ion at *m/z* 511 [M−H]^−^ yielded a predominant product ion at *m/z* 331 arising from the loss of a unit with a molecular weight of 180 amu. In positive ion mode, the protonated molecular ion of the compound did not appear, but its potassium adduct ion at *m/z* 551 [M+K]^+^ was found in its MS spectrum. Unfortunately, this peak was not identified despite our efforts.

Peak 2 shows a retention time of 12.0 min with maximal UV absorption at 200 nm. This peak gave a [M−H]^−^ ion at *m/z* 495, a [M−H+HCOOH]^−^ ion at *m/z* 541 and a [2M−H]^−^ ion at *m/z* 991, respectively. The ion at *m/z* 495 generated a predominant fragment ion in its MS^2^ spectrum at *m/z* 333 [M−H−glucosyl]^−^. In positive ion mode, peak 2 produced a very weak [M+H]^+^ ion but yielded prominent ions at *m/z* 519 [M+Na]^+^ and 535 [M+K]^+^. Consequently, it was characterized as a glucosyldeoxyandrographolide.

**Table 1:** The results of LODs, LOQs, regression equations, correlation coefficients and linearity ranges of andrographolide and dehydroandrographolide.

Analyte	Regression Equation	Correlation Coefficient	Linearity Range (μg/mL)	LOD (μg/mL)	LOQ (μg/mL)
Andrographolide	*y* = 20427*x* − 4906.5	0.9999	3.91–250	0.06	0.21
Dehydroandrographolide	*y* = 15967*x* − 11707	0.9999	15.62–250	0.12	0.49

**Table 2:** Results of precision, repeatability and stability tests.

Analyte	Precision (RSD, %)		Repeatability (RSD, %)	Stability (RSD, %)
	Intra-day (*n* = 5)	Inter-day (*n* = 5)		
Andrographolide	0.08	2.28	1.13	1.07
Dehydroandrographolide	0.07	2.69	1.33	1.02

**Table 3:** Accuracy of HPLC-UV method for determination of andrographolide and dehydroandrographolide.

Analyte	Sample Weight (g)	Original (mg)	Spiked (mg)	Found (mg)	Recovery (%)	Mean Recovery (%)	RSD (%)
Andrographolide	0.1002	1.1373	1.0000	2.2264	108.92	107.02	1.52
	0.1015	1.1520	1.0000	2.2198	106.78		
	0.1019	1.1566	1.0000	2.2368	108.02		
	0.1023	1.1611	1.0000	2.2070	104.59		
	0.1034	1.1736	1.0000	2.2417	106.81		
Dehydroandrographolide	0.1002	0.8427	0.8000	1.6436	100.11	98.86	1.46
	0.1015	0.8536	0.8000	1.6423	98.58		
	0.1019	0.8570	0.8000	1.6616	100.57		
	0.1023	0.8603	0.8000	1.6409	97.57		
	0.1034	0.8696	0.8000	1.6491	97.44		

**Table 4:** Contents of andrographolode and dehydroandrographolide in AH samples and similarity values of each sample compared to the reference fingerprint generated.

Sample No.	Content ^a^ (%)			Similarity			
				Correlation Coefficient		Angle Cosin	
	Andrographolide	Dehydroandrographolide	Total	Mean Value ^b^	Median Value ^c^	Mean Value	Median Value
AH-01	1.2504	0.9214	2.1718	0.9953	0.9963	0.9964	0.9972
AH-02	1.1316	0.8053	1.9368	0.9937	0.9948	0.9952	0.9961
AH-03	0.8043	1.3472	2.1515	0.9422	0.9382	0.9590	0.9558
AH-04	1.2835	0.5493	1.8328	0.9601	0.9623	0.9688	0.9706
AH-05	0.2235	0.3991	0.6226	0.9177	0.9127	0.9412	0.9371
AH-06	1.1932	1.1167	2.3100	0.9998	0.9995	0.9998	0.9996
AH-07	0.7361	0.5217	1.2578	0.9916	0.9930	0.9939	0.9950
AH-08	0.1122	0.4195	0.5317	0.6526	0.6435	0.7605	0.7530
AH-09	0.9147	0.4269	1.3415	0.9669	0.9702	0.9739	0.9765
AH-10	1.5926	0.8157	2.4083	0.9759	0.9783	0.9794	0.9815

^a^ Calculated with dried AH samples. ^b^ The reference fingerprint was generated with mean values of the samples. ^c^ The reference fingerprint was generated with median vales of the samples.

**Table 5:** Assignment of the characteristic peaks in AH samples.

No.	RT (min)	UV (nm)	MS in Neg. Mode	MS^2^ in Neg. Mode	MS in Pos. Mode	MS^2^ in Pos. Mode	Assignment	References
1	7.4	225	511 [M−H]^−^	331	551 [M+K]^+^			
			493 [M−H−H_2_O]^−^					
			557 [M−H+HCOOH]^−^					
2	12.0	200	495 [M−H]^−^	333 [M−H−glucosyl]^−^	519 [M+Na]^+^		Glucosyl-deoxyandrographolide	
			541 [M−H+HCOOH]^−^		535 [M+K]^+^			
			991 [2M−H]^−^					
3	12.4	225	349 [M−H]^−^	331 [M−H−H_2_O]^−^	297 [M+H−3H_2_O]^+^		Andrographolide	[16,17,18,19,20,25]
			331 [M−H−H_2_O]^−^		315 [M+H−2H_2_O]^+^			
			395 [M−H+HCOOH]^−^		333 [M+H−H_2_O]^+^			
4	12.7	251	495 [M−H]^−^	333 [M−H−glucosyl]^−^	519 [M+Na]^+^		Glucosyl-deoxyandrographolide	
			541 [M−H+HCOOH]^−^		535 [M+K]^+^			
5	12.9	287	495 [M−H]^−^	333 [M−H−glucosyl]^−^	519 [M+Na]^+^		Glucosyl-deoxyandrographolide	
			541 [M−H+HCOOH]^−^		535 [M+K]^+^			
6	13.1	227	349 [M−H]^−^	331 [M−H−H_2_O]^−^	315 [M+H−2H_2_O]^+^		Isoandrographolide	[17,25]
			331 [M−H−H_2_O]^−^					
			395 [M−H+HCOOH]^−^					
7	13.4	264	347 [M−H]^−^		349 [M+H]^+^		14-deoxy-11-oxoandrographolide	[16]
					313 [M+H−2H_2_O]^+^			
					331 [M+H−H_2_O]^+^			
8	16.6	201	479 [M−H]^−^	317 [M−H−glucosyl]^−^	481 [M+H]^+^	319 [M+H−glucosyl]^+^	Neoandrographolide	[16,17,18,19,25]
			525 [M−H+HCOOH]^−^		301 [M+H−glucosyl−H_2_O]^+^			
			959 [2M−H]^−^		319 [M+H−glucosyl]^−^			
					503 [M+Na]^+^			
					519 [M+K]^+^			
9	19.3	200	333 [M−H]^−^	305 [M−H−CO]^−^	335 [M+H]^+^	299 [M+H−2H_2_O]^+^	Deoxyandrographolide	[16,18,19,25]
					299 [M+H−2H_2_O]^+^	317 [M+H−H_2_O]^+^		
					317 [M+H−H_2_O]^+^			
10	19.8	251	331 [M−H]^−^	303 [M−H−CO]^−^	333 [M+H]^+^		Dehydroandrographolide	[16,19,20,25]
					297 [M+H−2H_2_O]^+^			
					315 [M+H−H_2_O]^+^			

Peak 3 occurs at a retention time of 12.4 min with maximal UV absorption at 225 nm. Ions at *m/z* 349, 331 and 395 were observed in its MS spectrum in negative ion mode, which were assigned as [M−H]^−^, [M−H−H_2_O]^−^ and [M−H+HCOOH]^−^ ions, respectively. An ion at *m/z* 331 [M−H−H_2_O]^−^ was found in its MS^2^ spectrum. The protonated molecular ion was not found in its MS spectrum in positive mode, but fragment ions corresponding to the loss of a series of water molecules at *m/z* 297 [M+H−3H_2_O]^+^, 315 [M+H−2H_2_O]^+^, and 333 [M+H−H_2_O]^+^ were found to be predominant ones. Based on the MS data reported in publications [16,17,18,19,20,25] and the comparison of its MS behaviors with that obtained from the reference compound, it was unequivocally identified as andrographolide.

Peak 4 and Peak 5 occur at retention times of 12.7 min and 12.9 min with maximal UV absorptions at 251 nm and 287 nm, respectively. In negative ion ESI experiments, they both yielded prominent deprotonated molecular ions at *m/z* 495 [M−H]^−^ and 541 [M−H+HCOOH]^−^. The MS^2^ spectrum of the ion at *m/z* 495 showed a characteristic ion at *m/z* 333 [M−H−glucosyl]^−^. In positive ion mode, the protonated molecular ion was not found, but the sodium and potassium adduct ions at *m/z* 519 [M+Na]^+^ and 535 [M+K]^+^ were observed as predominant ones. Each of the two peaks was identified as a glucosyldeoxyandrographolide.

Peak 6 shows a retention time of 13.1 min with maximal UV absorption at 227 nm. Characteristic ions at *m/z* 349 [M−H]^−^, 331 [M−H−H_2_O]^−^ and 395 [M−H+HCOOH]^−^ were produced from this peak in the MS spectrum in negative ion mode. The deprotonated ion at *m/z* 349 [M−H]^−^ gave a predominant ion at *m/z* 331 in the MS^2^ spectrum resulting from the loss of a neutral molecule of H_2_O. In positive ion mode, we did not find the protonated molecular ion, but it yielded a predominant ion at *m/z* 315 [M+H−2H_2_O]^+^ by losing two water molecules. According to the data in publications [17,25], the peak was identified as isoandrographolide.

Peak 7, with a maximal UV absorption at 264 nm, was eluted at a retention time of 13.4 min, and produced a [M−H]^−^ ion at *m/z* 347 in the MS spectrum in negative ion mode. In positive ion mode, the peak yielded a protonated molecular ion at *m/z* 349 [M+H]^+^ along with ions at *m/z* 313 [M+H−2H_2_O]^+^ and 331 [M+H−H_2_O]^+^. According to the literature data [16], the peak was identified as 14-deoxy-11-oxoandrographolide. 

Peak 8 was eluted at a retention time of 16.6 min with a maximal UV absorption at 201 nm. DA deprotonated molecular ion at *m/z* 479 [M−H]^−^, formic acid adduct ion at *m/z* 525 [M−H+HCOOH]^−^ and an ion at *m/z* 959 [2M−H]^−^ were observed in its MS spectrum in negative ion mode. The deprotonated ion at *m/z* 497 [M−H]^−^ gave a predominant ion at *m/z* 317 in the MS^2^ spectrum resulting from the loss of a glucosyl unit. In positive ion mode, a protonated molecular ion at *m/z* 481 [M+H]^+^ and its fragment ions at *m/z* 301 [M+H−glucosyl−H_2_O]^+^, 319 [M+H−glucosyl]^+^ as well as its sodium adduct ion at *m/z* 503 [M+Na]^+^ and potassium adduct ion at *m/z* 519 [M+K]^+^ were observed. A predominant ion at *m/z* 319 [M+H−glucosyl]^+^ was also found in its MS^2^ spectrum in positive ion mode. Based on the MS data reported in [16,17,18,19,25], it was identified as neoandrographolide. 

Peak 9 shows a retention time of 19.3 min and maximal UV absorption at 200 nm. This peak gave a [M−H]^−^ ion at *m/z* 333 in its MS spectrum and a [M−H−CO]^−^ ion at *m/z* 305 in its MS^2^ spectrum in negative ion mode. In positive ion mode, it produced a very week [M+H]^+^ ion at *m/z* 335, but yielded prominent ions at *m/z* 299 [M+H−2H_2_O]^+^ and *m/z* 317 [M+H−H_2_O]^+^, which were also the characteristic ions in its MS^2^ spectrum. Consequently, it was characterized as deoxyandrographolide [16,18,19,25].

Peak 10 occurs at a retention time of 19.8 min with maximal UV absorption at 251 nm. An ion at *m/z* 331 [M−H]^−^ was observed in its MS spectrum in negative ion mode, which yielded the characteristic ion at *m/z* 303 [M−H−CO]^−^. A protonated molecular ion at *m/z* 333 [M+H]^+^ and its fragment ions resulting from the loss of one or two water molecules at *m/z* 315 [M+H−H_2_O]^+^ and 297 [M+H−2H_2_O]^+^ appeared in its MS spectrum in positive ion mode. Based on the MS data reported in [16,19,20,25] and the comparison of its MS behavior with that obtained from the reference compound, it was unequivocally identified as dehydroandrographolide.

### 2.7. Fingerprinting and Chemometrics Analyses

Although the quantification results can confirm the contents of andrographolide and dehydroandrographolide in an AH sample, there is no way to know intuitively how similar an AH sample is to another one on the whole. Fingerprinting and chemometrics analyses, on the other hand, can show the chemical similarities between one and another one holistically and visually. On the one hand, the similarity of each chromatogram to the reference one (generated from all the AH samples) was calculated to show the similarities/differences between the samples. On the other hand, principal component analysis on the basis of the contents of andrographolide and dehydroandrographolide and PA/W values of the remaining eight characteristic peaks was performed. This operation can be thought of as revealing the internal structure of the data in a way which best explains the variance.

In fingerprinting analysis, 10 peaks shown in the overlapped chromatograms (Figure 1) were assigned as common peaks. The similarity values of the samples are listed in Table 4. Firstly, in all the samples, the similarity values of AH-08 were the lowest (below 0.80). Looking at the chromatogram of AH-08, we found that the characteristic peaks were smaller than those obtained from other AH samples. Furthermore, the lowest content of andrographolide and the second lowest content of dehydroandrographolide were found in AH-08. These two points mainly account for its lowest similarity values among all the samples. Secondly, the similarity values of AH-03 and AH-05 were between 0.91 and 0.96, and the ones of AH-04, AH-09 and AH-10 were between 0.96 and 0.99. These values were in the middle interval. Thirdly, four samples, AH-01, AH-02, AH-06 and AH-07 had similarity values higher than 0.99, indicating that their chemical profiles were very similar to that of the generated reference fingerprint. 

**Figure 2:**
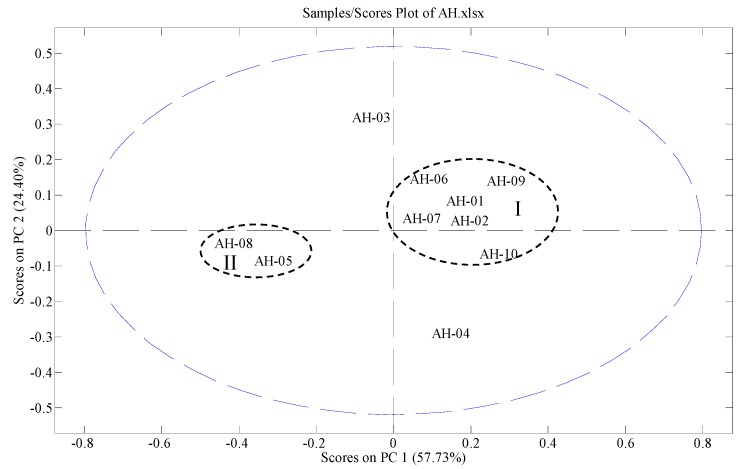
PCA scores plot of AH samples.

Except for andrographolide and dehydroandrographolide, although other seven peaks were identified, due to the unavailability of reference compounds, they were not quantified. Therefore, the variables of each sample in PCA consisted of the contents of andrographolide and dehydroandrographolide and PA/W values of the rest peaks. The data were exported to Excel (Microsoft, Inc., Belleview, WA, USA) to form a two-dimensional matrix (ten samples *versus* ten variables) which was then exported to SOLO for PCA. A two-component (the first two components) model cumulatively accounted for 78.74% of total variance, based on which PCA scores plot (Figure 2) was generated. From the scores plot, we can see intuitively that AH-01, AH-02, AH-06, AH-07, AH-09 and AH-10 are clustered tightly in group I. They all get PC1 scores higher than zero. AH-05 and AH-08 are clustered in group II. They both get PC1 scores lower than zero. AH-03 and AH-04 are located relatively far away from the two groups. 

The distances between AH samples indicate the similarities/differences between their chemical profiles. Those samples with similar chemical profiles are near to each other. In the contrary, differences in chemical profiles lead to larger distances between the samples. To find out how variables contribute to the significant differences between different AH samples, PC1 and PC2 loadings plots were generated. The PC1 loadings plot indicates that peak 3 (andrographolide), peak 6 (isoandrographolide) and peak 9 (deoxyandrographolide) contribute positively to the positions of AH samples on PC1 significantly, whereas, peak 5 (glucosyldeoxyandrographolide) and peak 7 (14-deoxy-11-oxoandrographolide) contribute negatively in PC1. In detail, a higher content of peak 3 (andrographolide) and PA/W values of peak 6 (isoandrographolide) and peak 9 (deoxyandrographolide) lead to higher PC1 scores of a sample in the scores plot, moving its position to the right. Meanwhile, higher PA/W values of peak 5 (glucosyldeoxyandrographolide) and peak 7 (14-deoxy-11-oxoandrographolide) lead to lower PC1 scores of a sample. In our study, AH-10 has the highest content of peak 3 (andrographolide). AH-01 has the highest PA/W values of peak 6 (isoandrographolide) and peak 9 (deoxyandrographolide). AH-02 shows the second highest PA/W values of peak 6 (isoandrographolide) and peak 9 (deoxyandrographolide). Therefore, they are in the rightmost positions following closely after AH-09 which has the lowest PA/W values of peak 5 (glucosyldeoxyandrographolide) and peak 7 (14-deoxy-11-oxoandrographolide). AH-05 was found to have the highest PA/W value of peak 7 (14-deoxy-11-oxoandrographolide) and the lowest PA/W value of peak 9 (deoxyandrographolide). AH-08 was found to have the lowest content of peak 3 (andrographolide). Therefore, the two samples are placed in the leftmost position clustering in group II.

According to the PC2 loadings plot, peak 1, peak 2 (glucosyldeoxyandrographolide), peak 8 (neoandrographolide) and peak 10 (dehydroandrographolide) mainly influence the position of each sample in PC2. Peak 2 (glucosyl-deoxyandrographolide), peak 8 (neoandrographolide) and peak 10 (dehydroandrographolide) contribute to the PC2 positions of AH samples positively, whereas, peak 1 contributes negatively. AH-03 has the highest content of dehydroandrographolide (peak 10) and the highest PA/W values of peak 2 (glucosyldeoxyandrographolide) and peak 8 (neoandrographolide), making it locate on the top in the scores plot. AH-04 was found to have the highest PA/W value of peak 1, resulting in its lowest position in the scores plot. All in all, the scores plot shows the distributions of the tested samples intuitively and clearly, whereas, loadings plots indicate the influences of the variables on the positions of AH samples in the scores plot.

### 2.8. Antioxidant Activity of AH Samples

#### 2.8.1. DPPH Assay

**Figure 3:**
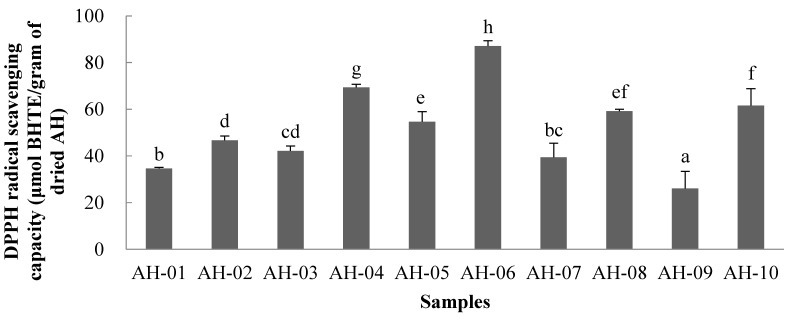
DPPH radical scavenging capacity of AH samples. The values are expressed as μmol BHT equivalents per gram dried AH sample (mean ± SD). Values that share the same letter are not significantly different (*p* < 0.05).

The DPPH free radical scavenging activities of the AH samples are shown in Figure 3. For each sample, three concentrations were tested. AH-06 had the highest value at 87.01 μmol BHTE/g dried sample, followed by AH-04 at 69.28 μmol BHTE/g dried sample.

**Figure 4:**
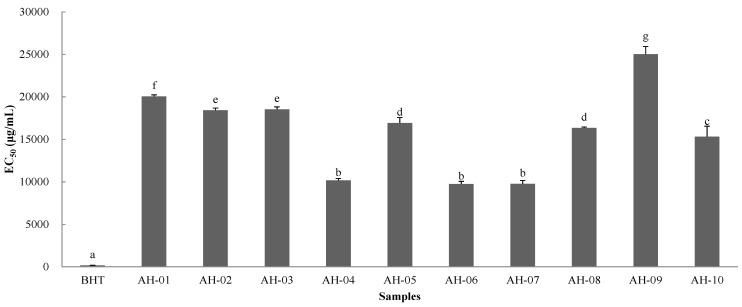
EC_50_ values of BHT and AH samples. Values that share the same letter are not significantly different (*p* < 0.05).

AH-09 displayed the lowest value at 26.07 μmol BHTE/g dried sample. In order to quantify the antioxidant activity further, EC_50_ values were calculated (Figure 4). Although the DPPH radical-scavenging activities of all the AH samples were weaker than that of BHT, the results showed the different scavenging power of the samples.

#### 2.8.2. FRAP Assay

**Figure 5:**
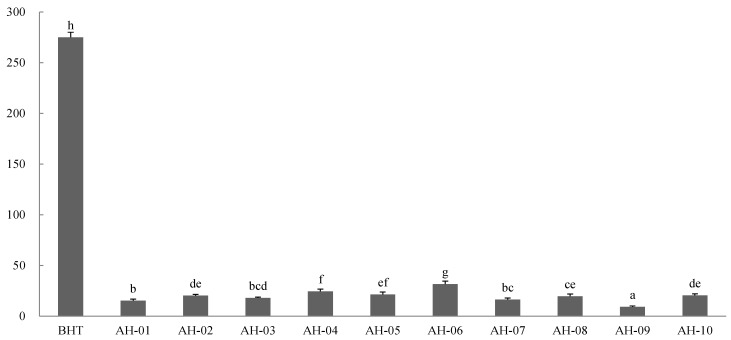
Ferric reducing capacity of AH samples. The values are expressed as μmol Fe^2+^ equivalents per gram of dried AH sample (mean ± SD). Values that share the same letter are not significantly different (*p* < 0.05).

Being expressed as Fe^2+^ equivalents, the FRAP values were applied to determine the ferric reducing capacities of the AH samples (Figure 5). The values were all between 9.28 (AH-09) and 31.82 (AH-06) μmol Fe^2+^ equivalents/g dried AH sample. BHT showed the highest value at 275.01 μmol Fe^2+^ equivalents/g BHT. Similar to the results obtained from the DPPH assay, the ferric reducing capacities of all the AH samples were weaker than that of BHT, however, the differences between the samples were significant.

#### 2.8.3. Correlation Analysis

Spearman correlation was calculated using bivariate correlation analysis since just ten AH samples were tested in the present study and we were unable to judge whether they obeyed a Gaussian distribution or not. The results are shown in Table 6 which indicate that peak 1, peak 3, peak 4, peak 5, peak 7 and peak 10 have a positive correlation relationship to DPPH free radical scavenging and ferric reducing capacities, whereas, peak 2, peak 6, peak 8 and peak 9 are negatively correlated to DPPH free radical scavenging and ferric reducing capacities.

**Table 6:** Spearman’s correlation between the characteristic peaks of each AH sample and its antioxidant capacities.

Peak No.	DPPH Free Radical Scavenging Activity	Ferric Reducing Capacity
	Spearman’s Correlation	Spearman’s Correlation
Peak 01	0.236	0.236
Peak 02	−0.127	−0.127
Peak 03	0.285	0.261
Peak 05	0.297	0.370
Peak 06	−0.018	0.018
Peak 07	0.212	0.297
Peak 08	−0.503	−0.467
Peak 09	−0.224	−0.261
Peak 10	0.115	0.079

## 3. Experimental Section

### 3.1. Chemicals, Solvents and Herbal Materials

Andrographolide and dehydroandrographolide were purchased from the Shanghai R & D Center for Standardization of Traditional Chinese Medicine (Shanghai, China). LC-grade methanol, acetonitrile were purchased from the Merck Co. branch in Taipei, Taiwan. Purified water was prepared with a Milli-Q system (Millipore, Milford, MA, USA). All other reagents used in the present study were of analytical grade. Herbal AH materials were purchased from local pharmacies of Taiwan, and marked as AH-01 to AH-10. All the specimens have been deposited in Department of Chinese Pharmaceutical Sciences and Chinese Medicine Resources, School of Pharmacy, China Medical University.

### 3.2. Sample and Reference Preparation

AH samples were dried in the shade and were ground into a fine powder (20 mesh) using a grinder with a knife blade. Each AH powder sample (0.2 g) was carefully weighed into a 15 mL centrifuge tube. Methanol (10 mL) was then added to the tube and shaken briefly. Each sample was then extracted with an ultrasonic cleaner (Delta DC400H, Taiwan Delta New instrument Co., Taipei, Taiwan) at a frequency of 40 kHz at 25 °C for 30 min. The extract of each sample was centrifuged for 10 min at 3000 rpm and the supernatant was then transferred to a 25 mL volumetric flask. The procedure was repeated one more time and the supernatants were combined. The final volume was made up to 25 mL with methanol which was then filtered through a 0.45 μm PVDF syringer filter (VWR Scientific, Seattle, WA, USA) for analysis. The reference compounds of andrographolide and dehydroandrographolide were accurately weighed respectively and were dissolved in methanol both at 500 mg/L (stock solutions). The stock solutions were then diluted to appropriate concentrations for establishment of calibration curves. An aliquot of 10 μL of each solution was used for HPLC and HPLC-ESI-MS analyses.

### 3.3. HPLC Analysis

HPLC analyses were performed on a Waters 2695 HPLC system equipped with a Waters 2998 photodiode array detector (PDA, Waters Corporation, Milford, MA, USA), Waters e2695 separations module and column heater module. An XBridge^TM^ Shield RP 18 column (250 mm × 4.6 mm, 3.5 μm, Waters Corporation, Milford, MA, USA) was used. The mobile phase consisted of water (A) and acetonitrile (B). The optimized elution conditions were as follow: 0–25 min, 20%–55% B. The flow rate was 1 mL/min and the injection volume was 10 μL. UV spectra were acquired from 190 nm to 400 nm. The autosampler and column compartment were maintained at 25 °C and 35 °C, respectively.

### 3.4. HPLC-ESI-MS Analysis

HPLC-ESI-MS analyses were performed on a TSQ Quantum Access Max Triple Stage Quadrupole Mass Spectrometer (Thermo Fisher Scientific Inc., Waltham, MA, USA) with an Accela 1250 UHPLC system equipped with an Accela 1250 photo diode array (PDA) detector, an Accela HTC PAL autosampler, and an Accela 1250 binary pump. The column and elution conditions used were the same as that described in 3.3 HPLC analysis, except that the flow rate was set at 0.25 mL/min with a split ratio. Ultrahigh pure helium (He) and high purity nitrogen (N_2_) were used as collision gas and nebulizer, respectively. The optimized parameters in negative/positive ion modes were as follows: ion spray voltage, −2.5 kV/3.0 kV; auxiliary gas, 40 arbitrary units; sheath gas, 15 arbitrary units; capillary temperature, 350 °C; vaporizer temperature, 350 °C; capillary offset, −35V/35 V. Spectra were recorded in the range of *m/z* 100–1500 for full scan data, meanwhile, the normalized collision energy was tested from 25% to 45% for MS^2^ data using dependant scan. 

### 3.5. Antioxidant Activities 

#### 3.5.1. DPPH Assay

The DPPH free radical-scavenging activity of each sample was assayed according to the established methods in published articles [26,27,28] with minor modifications. In brief, a 0.3 mM solution of methanolic DPPH was freshly prepared. An aliquot (50 μL) of each sample (with appropriate enrichment or dilution if necessary) was added to methanolic DPPH solution (150 μL) to initiate the reaction. Discolorations were measured at 517 nm after reaction for 30 min at room temperature in the dark. Measurements were performed in triplicate. EC_50_ value calculated denotes the concentration of one sample required to decrease the absorbance by 50%. The antioxidant capacity was expressed as μmol BHT equivalents/gram of dried AH sample.

#### 3.5.2. FRAP Assay

The ability to reduce ferric ions was measured based on the methods reported in previous papers [28,29] with minor modifications. Briefly, each sample (30 μL, with appropriate enrichment or dilution if necessary) was added to FRAP reagent (200 μL, 10 parts of 300 mM sodium acetate buffer at pH 3.6, 1 part of 10.0 mM TPTZ solution, and 1 part of 20.0 mM FeCl_3_·6H_2_O solution), and the reaction mixture was kept at room temperature for 5 min. Fresh working solutions of FeSO_4_·7H_2_O were used for calibration. The antioxidant capacity of reducing ferric ions of each sample was expressed as μmol Fe^2+^ equivalents/gram of dried AH sample.

### 3.6. Data Analysis

#### 3.6.1. Similarity Analysis

Similarity values of the chromatographic fingerprints of AH samples were calculated using the following two formulas:

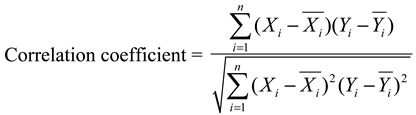
(1)

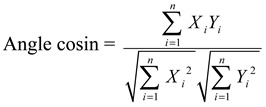
(2)
where *X_i_* and *Y_i_* represented the peak area of the characteristic peak in each sample and the peak area of the characteristic peak in reference fingerprint generated, respectively.

#### 3.6.2. PCA

The data obtained from chromatographic fingerprints were analyzed with Solo (Eigenvector Research, Inc., Wenatchee, WA, USA). Normalization (2-Norm, length = 1) and mean center were used for data reprocessing before PCA was performed.

#### 3.6.3. One-way ANOVA and Correlation Analyses

One-way ANOVA on the data obtained from antioxidant assays and correlation analysis between the ten characteristic peaks and their antioxidant capacities were calculated using SPSS Statistics 17.0 (SPSS Inc., Chicago, IL, USA) 

## 4. Conclusions

An HPLC-UV-MS method was developed and validated after detailed investigation on extraction of chemical compounds from AH using different solvents and extraction times. Regarding the ten characteristic peaks, andrographolide and dehydroandrographolide were quantified accurately, and the remaining peaks (except peak 1) were identified. Based on the contents and PA/W values of the characteristic peaks, PCA scores plot and loadings plots were obtained. The similarity values of the tested samples were seen visually from the scores plot, meanwhile, how the values (variables in PCA) contribute to the distribution of the samples were explained via PC1 and PC2 loadings plots. Furthermore, DPPH free radical-scavenging and ferric reducing capacities of the AH samples were assayed. Based on the obtained data, how the characteristic peaks correlate to the antioxidant capacities were demonstrated via bivariate correlation analysis for the first time.

## Supplementary Materials

Supplementary materials can be accessed at: http://www.mdpi.com/1420-3049/19/11/18332/s1.

## Supplementary Files

Supplementary File 1
