# Supplementary material for: Chemical Compositions, Chromatographic Fingerprints and Antioxidant Activities of Andrographis Herba
Source: Molecules. 2014 Nov 10;19(11):18332–50. doi: 10.3390/molecules191118332 (PMC6270826; doi:10.3390/molecules191118332)
Supplement: Supplementary File 1 [file molecules-19-18332-s001.pdf]

Supplementary Materials

Figure S1. Chromatograms of AH-01 extracted using ethanol for three times and the peak areas of andrographolide and dehydroandrographolide.

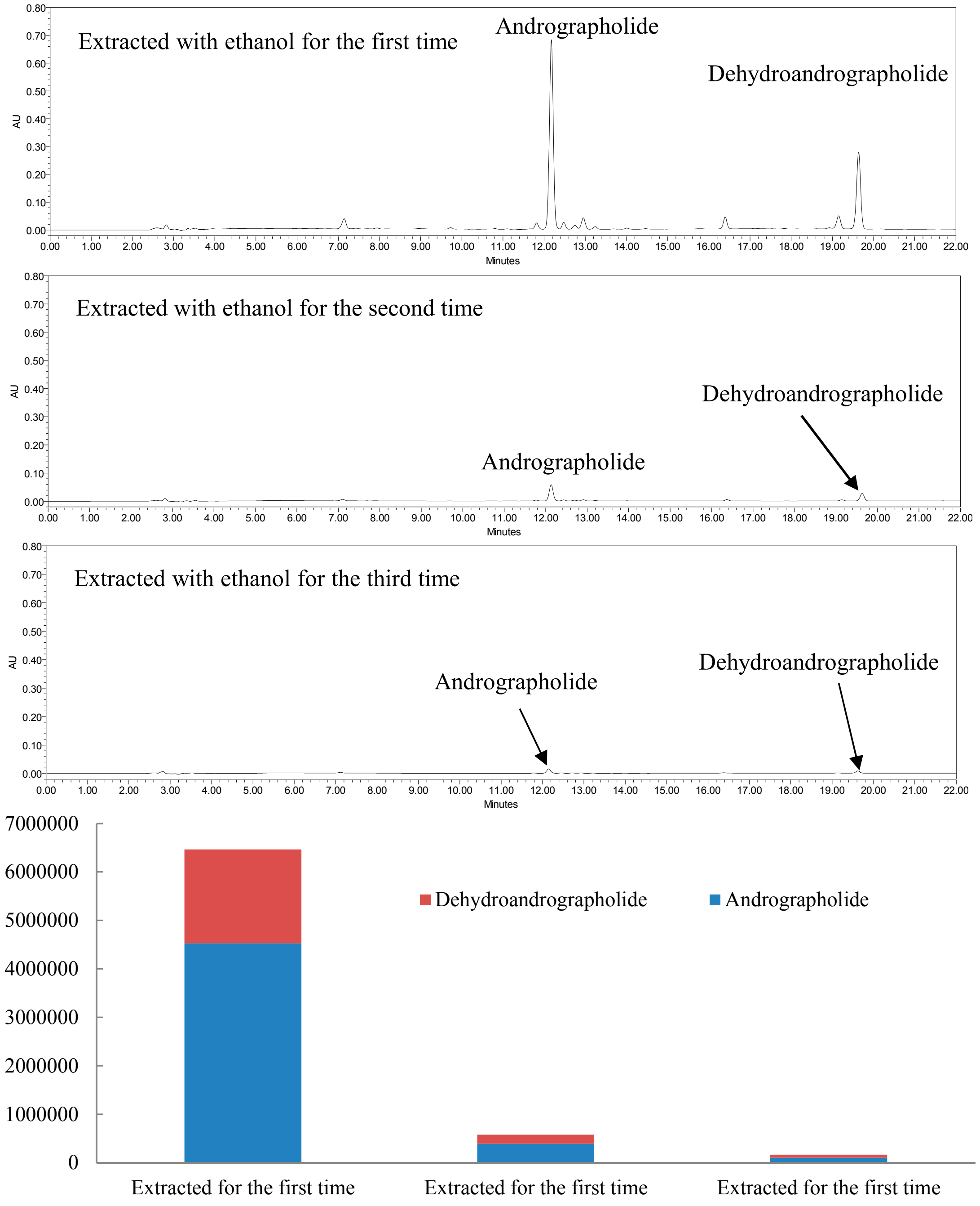

Figure S2. Chromatograms of AH-01 extracted using methanol for three times and the peak areas of andrographolide and dehydroandrographolide.

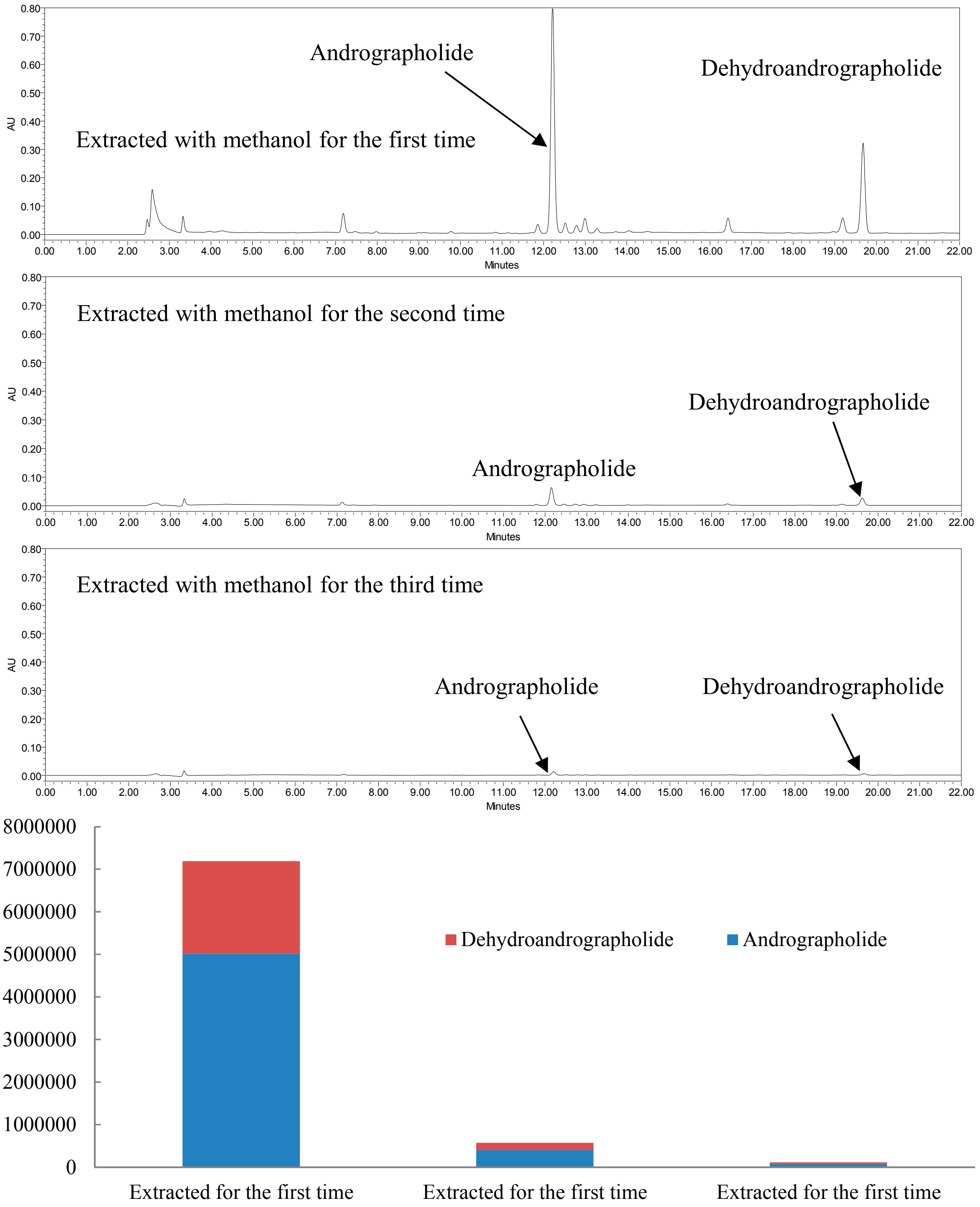

Table S1. RSDs of relative retention times and relative peak areas of the ten characteristic peaks.

Peak No.	Inter-day Precision		Intra-day Precision		Repeatability		Stability	
	RRT	RPA	RRT	RPA	RRT	RPA	RRT	RPA
1	0.12	0.99	0.33	2.07	0.00	3.03	0.01	1.22
2	0.02	1.66	0.01	1.90	0.07	1.72	0.15	3.77
3	0.00	0.00	0.00	0.00	0.00	0.00	0.00	0.00
4	0.02	1.43	0.01	1.16	0.06	1.00	0.06	3.47
5	0.03	2.14	0.02	3.41	0.01	3.43	0.12	3.93
6	0.03	0.43	0.02	0.80	0.02	2.32	0.06	2.08
7	0.04	0.88	0.01	3.59	0.04	2.98	0.07	4.43
8	0.11	0.52	0.05	1.42	0.07	0.64	0.20	1.15
9	0.12	0.44	0.11	1.38	0.07	1.37	0.55	0.82
10	0.13	0.22	0.12	0.38	0.08	0.33	0.64	0.91

Table S2. Similarity values for the chromatographic fingerprinting method.

Parameters	Similarity				
	Value	Inter-day precision	Intra-day precision	Repeatability	Stability
Correlation coefficient	Mean value	>0.99	>0.99	>0.99	>0.99
	Median value	>0.99	>0.99	>0.99	>0.99
Angle cosin	Mean value	>0.99	>0.99	>0.99	>0.99
	Median value	>0.99	>0.99	>0.99	>0.99
